# Supplementary material for: Expectation generation and its effect on subsequent pain and visual perception
Source: bioRxiv. 2024 Oct 12:2024.10.10.617570. Preprint. [Version 1] doi: 10.1101/2024.10.10.617570 (PMC11482957; doi:10.1101/2024.10.10.617570)
Supplement: Supplement 1 [file NIHPP2024.10.10.617570v1-supplement-1.pdf]

bioRxiv preprint doi: https://doi.org/10.1101/2024.10.10.617570; this version posted October 12, 2024. The copyright holder for this preprint (which was not certified by peer review) is the author/funder, who has granted bioRxiv a license to display the preprint in perpetuity. It is made available under aCC-BY 4.0 International license.

Supplementary Information

Expectation generation and its effect on subsequent pain and visual perception

Rotem Botvinik-Nezer[1,2,*], Stephan Geuter[3], Martin A. Lindquist[3] and Tor D. Wager[2,*]

[1]Hebrew University of Jerusalem; [2]Dartmouth College; [2]Johns Hopkins University;

[*]Corresponding authors

bioRxiv preprint doi: https://doi.org/10.1101/2024.10.10.617570; this version posted October 12, 2024. The copyright holder for this preprint (which was not certified by peer review) is the author/funder, who has granted bioRxiv a license to display the preprint in perpetuity. It is made available under aCC-BY 4.0 International license.

Supplementary Results

Computational Modeling of Perception

We developed five computational models to test whether and how cue-based expectations are weighted when performing perception, whether the effect of expectations persist, and whether these processes are the same or different across modalities. In all models, expectations were computed for each trial of each participant based on the cue, with the optimized participant-level model from the expectation data. The intensity-based (expectation-independent) rating of each stimulus was determined based on the mean rating of each participant for a given intensity in the stimulus-response task, with a free parameter s per modality (s_p for pain and s_v for vision) that serves as a linear scaling factor, to account for the different context between the stimulus-response and cued-perception tasks (e.g., outside vs. inside the MRI scanner) and physiological processes (e.g., habituation or differences in sensitivity across skin sites).

The Baseline Model assumes the cue-based expectations do not affect perception throughout the task, and thus the rating is only based on the stimulus intensity and the scaling factor. In the Expectation Model, there are significant cue effects, and perception is based on a combination of the stimulus intensity and the cue-based expectations, with a free parameter w which is fixed throughout the task for each participant. This parameter determines the weight given to cue-based expectations vs. incoming information (stimulus intensity). In the "Expectation Learning Model", there are also significant cue effects, and the relative contribution of expectation vs. stimulus intensity at the beginning of the task is determined by the free parameter $w0$. Then, w is updated from trial to trial based on prediction errors between the expected and empirical rating, with learning rate α. The two last models are equivalent to the second and third models, but include separate free parameters for each modality. In the "Expectation Model by Modality", w_p and w_v model the weighting of expectations for pain and visual perception trials, respectively. In the "Expectation Learning Model by Modality", $w0_p$ and $w0_v$ model the initial weighting of the expectation in each modality, and α_p and α_v model the learning rate for the pain and visual perception, respectively.

We fit each model to each participant's data from the cued-perception task, and optimized the free parameters based on ordinal least squares between the predicted and empirical ratings of the

bioRxiv preprint doi: https://doi.org/10.1101/2024.10.10.617570; this version posted October 12, 2024. The copyright holder for this preprint (which was not certified by peer review) is the author/funder, who has granted bioRxiv a license to display the preprint in perpetuity. It is made available under aCC-BY 4.0 International license.

painfulness or visual contrast of the stimulus. We also compared the five models and selected the best model for each participant based on F tests for nested models. Of 45 participants, the Baseline Model was the best model for 8 participants (18% of sample), the Expectation Model was best for 16 participants (35%), the Expectation Learning Model was best for 5 participants (11%), the Expectation Model by Modality was best for 8 participants (18%), and the Expectation Learning Model by Modality was best for 8 participants (18%).

In line with the participant-level results, group-level F tests for nested models based on the residual sum of squares indicated that the Expectation Model fits the data significantly better than the Baseline model (F = 12.40, p = .001), but the more complex models do not significantly improve the fit (Expectation Model vs. Expectation Learning Model: F = 2.04, p = .161; Expectation Model vs. Expectation Model by Modality: F = 1.06, p = .309; Expectation Model by Modality vs. Expectation Learning Model by Modality: F = 1.47, p = .242). Comparison based on the Akaike Information Criterion (AIC) indicated that the Expectation Model and the Expectation Learning Model were the best models while accounting for complexity (AIC values: Baseline Model = 309.81; Expectation Model = 300.17; Expectation Learning Model = 299.99 Expectation Model by Modality = 301.02; Expectation Learning Model by Modality = 301.75). These results suggest that the cues affected participants' perception, and that only some participants learned to downweight (or ignore) the cues during the task. They further suggest that these processes were largely similar across modalities.

Finally, a direct comparison of w_p and w_v showed that participants weighted the cues more in pain than visual trials (paired t-test, mean difference = 0.090, *SD* difference = 0.191, *Cohen's d* = 0.471, $t_{(44)}$ = 3.162, p = .003), indicating greater influences of cues on pain perception than visual perception, though cue effects were strong in both modalities.

Effects on neural perceptual processing

In addition to the results reported in the main text, we further tested "off target" effects–stimulus intensity and cue effects on visual regions during pain perception, or pain regions during visual perception. Activity was higher (less negative) during more intense heat stimuli in several visual regions, probably because noxious stimuli produce diffuse effects, but none of them was affected

bioRxiv preprint doi: https://doi.org/10.1101/2024.10.10.617570; this version posted October 12, 2024. The copyright holder for this preprint (which was not certified by peer review) is the author/funder, who has granted bioRxiv a license to display the preprint in perpetuity. It is made available under aCC-BY 4.0 International license.

by the cue during pain perception. Activity in the right IPS was higher during pain perception following cues with lower mean (similar to the aversive PE-like activity in the PAG; $p = .047$). Conversely, activity during visual perception was not affected by the stimulus intensity in any of the pain processing regions (all ps $\geq .162$), but it was higher following cues with lower mean in the right VPL/VPM thalamus ($p = .046$), and lower following negatively skewed compared to symmetric cues in the right dorsal posterior insula ($p = .001$) and right S1 ($p = .018$). In addition, there was a significant cue mean x cue variance interaction effect on activity in the right NAc (shell-like) during visual perception ($p = .048$), such that activity was slightly more negative following high compared to low mean for low variance cues, but less negative for high compared to low mean for high variance cues (not in line with Bayesian predictive coding accounts).

Effects on anticipatory neural activity

While both the current study and most previous ones focused on cue effects on stimulus-evoked activity, we also performed an exploratory analysis to test anticipatory activity (see Supplementary Figure 3 and Supplementary Figure 4). Several pain processing regions were more active during anticipation in response to high vs. low cue mean, including the dorsal posterior insula (right $p = .030$; left $p = .039$), aMCC ($p = .008$), PAG ($p = .049$), and anterior insula (right $p = .023$; left $p = .010$). In addition, cue-evoked activity in the PAG ($p = .043$) and NAc (core-like; right $p = .020$; left $p = .049$) was lower following positively skewed compared to symmetric cues (opposite direction than expected and observed behaviorally, although it was not significant behaviorally). On visual trials, anticipatory activity was lower following high vs. low cue mean in V4 (right $p = .025$; left $p = .028$), left V5 ($p = .001$), left VMV2 ($p = .003$), left VMV3 ($p = .017$), left V3B ($p = .005$), left V4t ($p = .001$), IPS (right $p = .001$; left $p = .034$), and left dlPFC ($p = .046$). It was also higher for cues with higher variance (less certain cues) in right V4 ($p = .020$), left NAc (core-like, $p = .017$) and VPL/VPM thalamus (right $p = .046$, left $p = .038$). As for the skewness, anticipatory activity in visual trials was higher for negatively skewed compared to symmetric cues (opposite from the behavioral direction) in the right IPS ($p = .006$) and lower for positively skewed compared to symmetric cues (again, opposite from the behavioral effect) in the right NAc (core-like $p = .004$; shell-like $p = .018$). Finally, there was a significant interaction between the cue mean and the cue variance in the right VMV3 ($p = .042$), such that anticipatory activity was higher for high compared

bioRxiv preprint doi: https://doi.org/10.1101/2024.10.10.617570; this version posted October 12, 2024. The copyright holder for this preprint (which was not certified by peer review) is the author/funder, who has granted bioRxiv a license to display the preprint in perpetuity. It is made available under aCC-BY 4.0 International license.

to low cue mean for more certain cues, and higher for low compared to high cue mean for less certain cues (not in line with Bayesian predictive coding accounts).

bioRxiv preprint doi: https://doi.org/10.1101/2024.10.10.617570; this version posted October 12, 2024. The copyright holder for this preprint (which was not certified by peer review) is the author/funder, who has granted bioRxiv a license to display the preprint in perpetuity. It is made available under aCC-BY 4.0 International license.

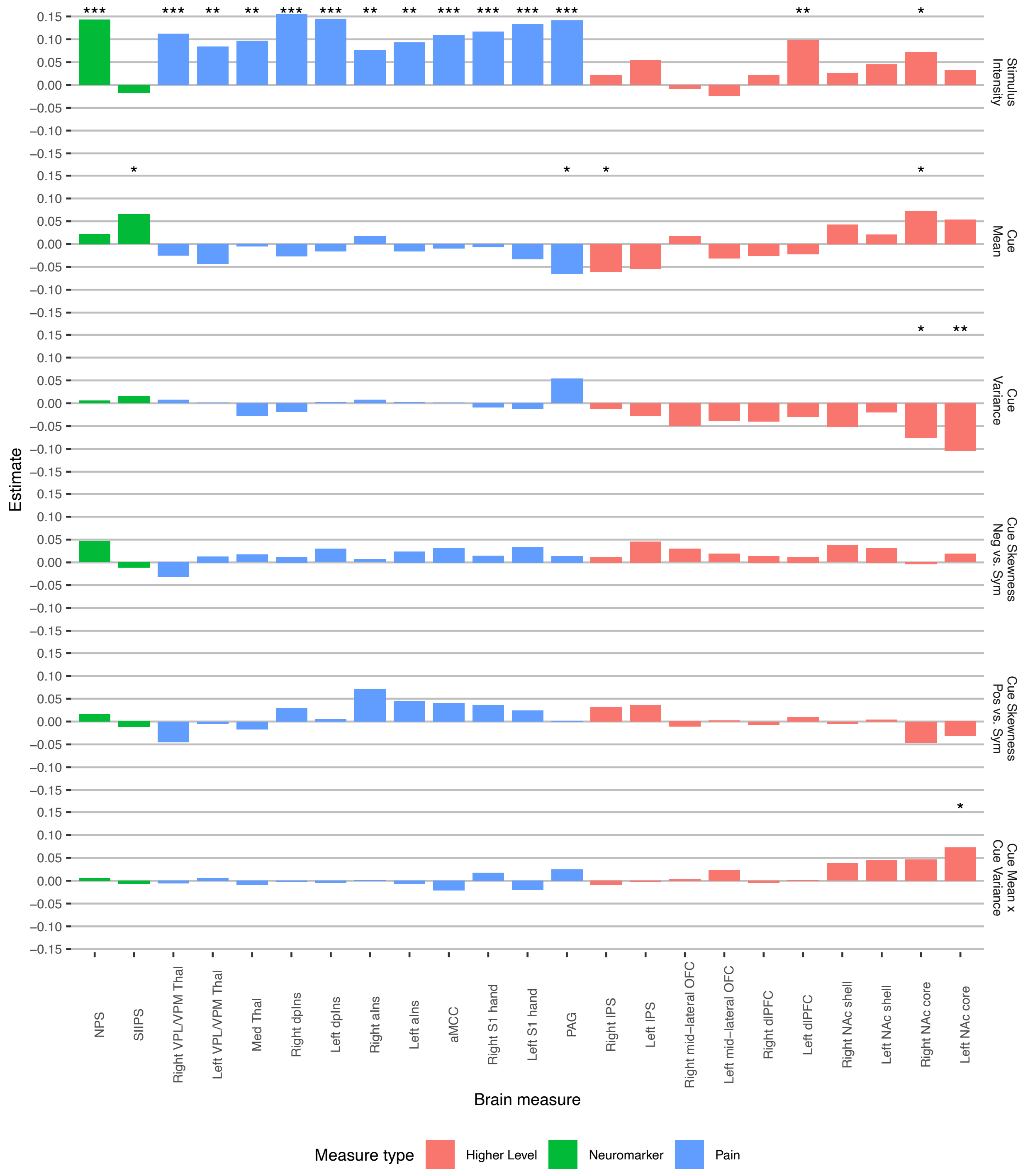

Supplementary Figure 1. Stimulus intensity and cue effects across all ROIs during thermal stimuli. Asterisks represent the level of significance (* $p < .05$, ** $p < .01$, *** $p < .001$). Abbreviations: Neg = negative; Pos = positive; Sym = symmetric; NPS = Neurological Brain Signature; SIIPS = Stimulus Intensity Independent Pain Signature; VPL/VPM = ventral posterior lateral/medial; Thal = thalamus; dpIns = dorsal posterior insula; aIns = anterior insula; aMCC = anterior midcingulate cortex; PAG = periaqueductal gray; IPS = intraparietal sulcus; OFC = orbitofrontal cortex; dlPFC = dorsolateral prefrontal cortex; NAc = nucleus accumbens.

bioRxiv preprint doi: https://doi.org/10.1101/2024.10.10.617570; this version posted October 12, 2024. The copyright holder for this preprint (which was not certified by peer review) is the author/funder, who has granted bioRxiv a license to display the preprint in perpetuity. It is made available under aCC-BY 4.0 International license.

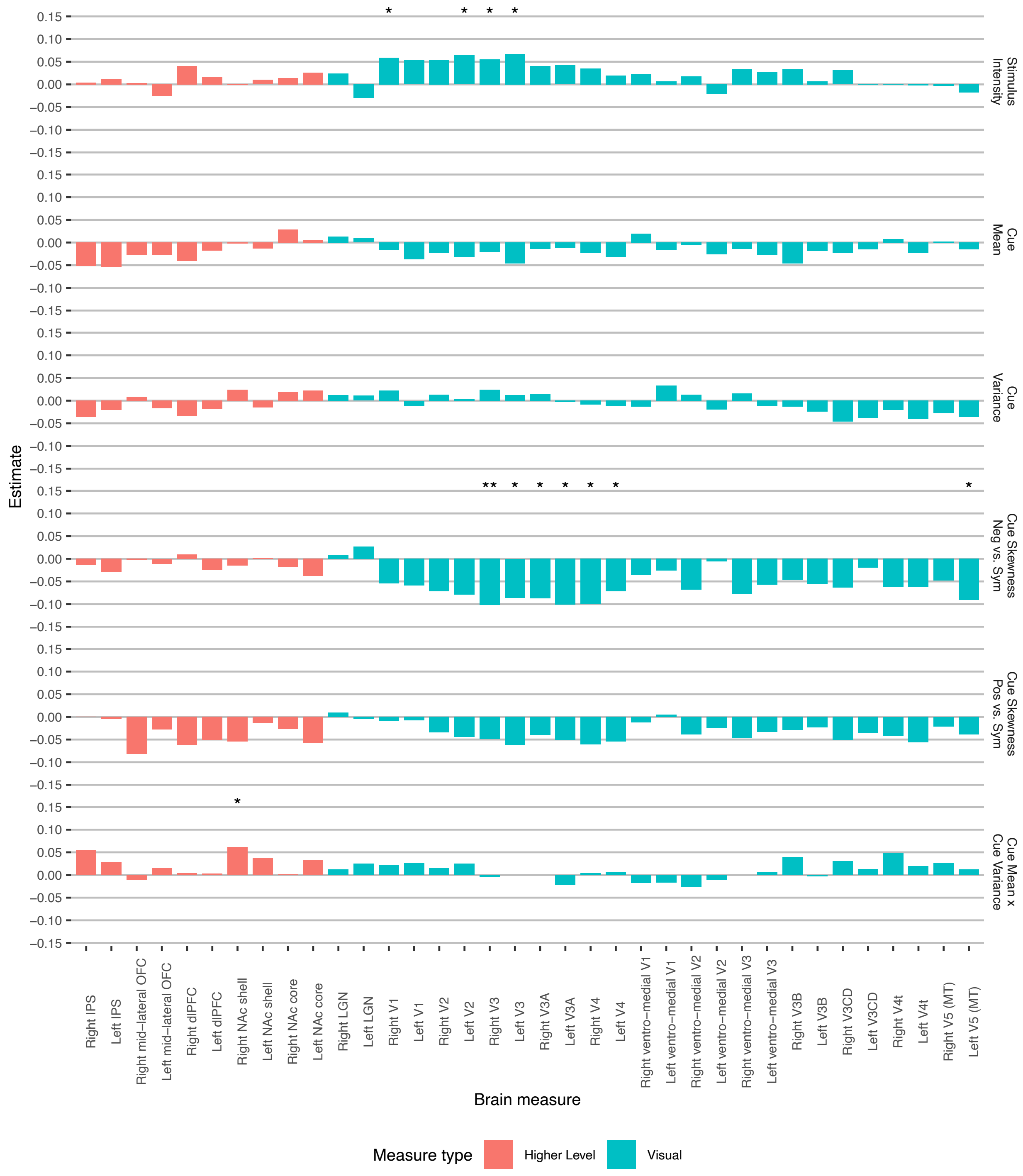

Supplementary Figure 2. Stimulus intensity and cue effects across all ROIs during visual stimuli. Asterisks represent the level of significance (* $p < .05$, ** $p < .01$, *** $p < .001$). Abbreviations: Neg = negative; Pos = positive; Sym = symmetric; IPS = intraparietal sulcus; OFC = orbitofrontal cortex; dlPFC = dorsolateral prefrontal cortex; NAc = nucleus accumbens; LGN = lateral geniculate nucleus.

bioRxiv preprint doi: https://doi.org/10.1101/2024.10.10.617570; this version posted October 12, 2024. The copyright holder for this preprint (which was not certified by peer review) is the author/funder, who has granted bioRxiv a license to display the preprint in perpetuity. It is made available under aCC-BY 4.0 International license.

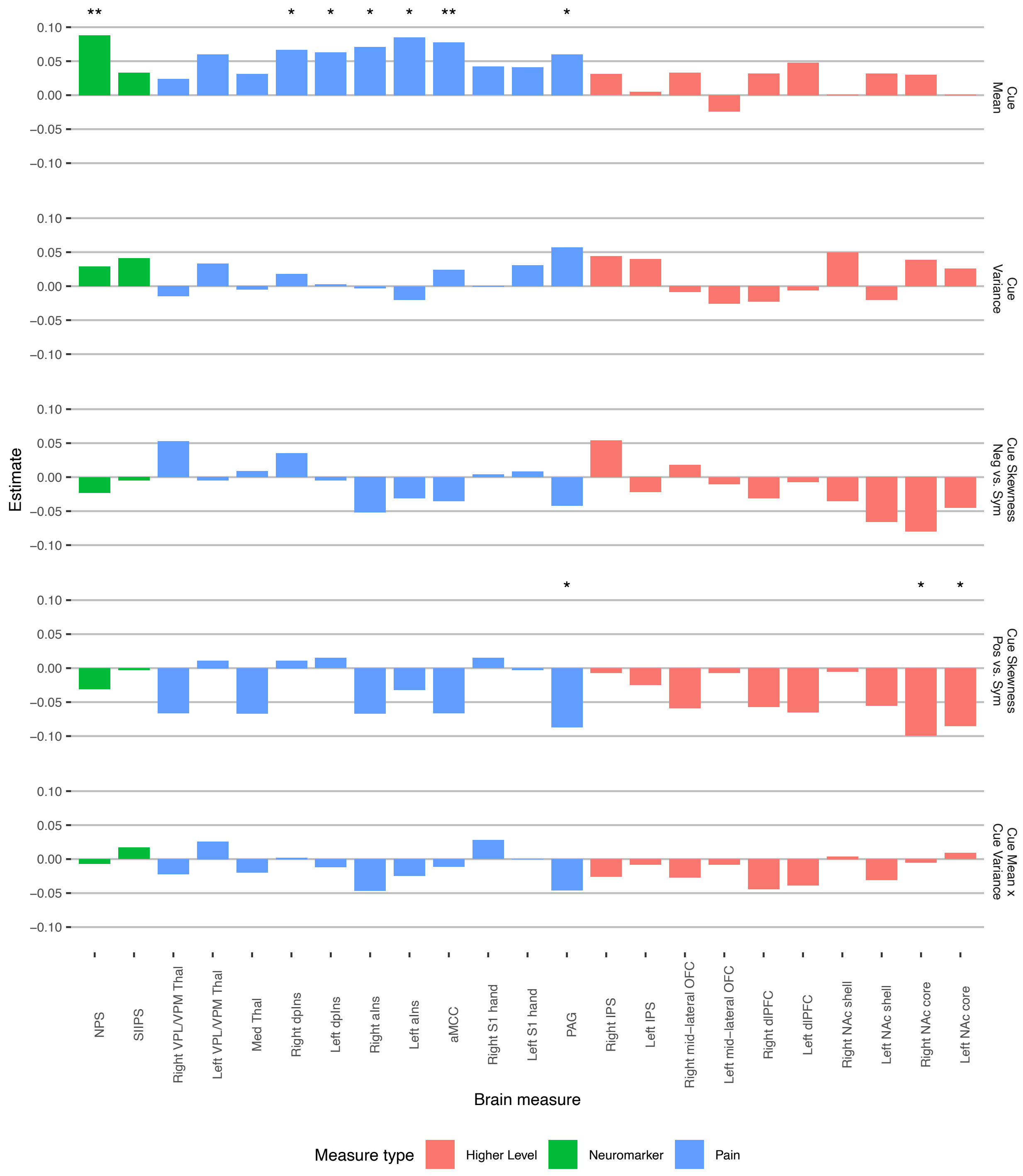

Supplementary Figure 3. Cue effects across all ROIs during anticipation for thermal stimuli. Asterisks represent the level of significance (* $p < .05$, ** $p < .01$, *** $p < .001$). Abbreviations: Neg = negative; Pos = positive; Sym = symmetric; NPS = Neurological Brain Signature; SIIPS = Stimulus Intensity Independent Pain Signature; VPL/VPM = ventral posterior lateral/medial; Thal = thalamus; dpIns = dorsal posterior insula; aIns = anterior insula; aMCC = anterior midcingulate cortex; PAG = periaqueductal gray; IPS = intraparietal sulcus; OFC = orbitofrontal cortex; dlPFC = dorsolateral prefrontal cortex; NAc = nucleus accumbens.

bioRxiv preprint doi: https://doi.org/10.1101/2024.10.10.617570; this version posted October 12, 2024. The copyright holder for this preprint (which was not certified by peer review) is the author/funder, who has granted bioRxiv a license to display the preprint in perpetuity. It is made available under aCC-BY 4.0 International license.

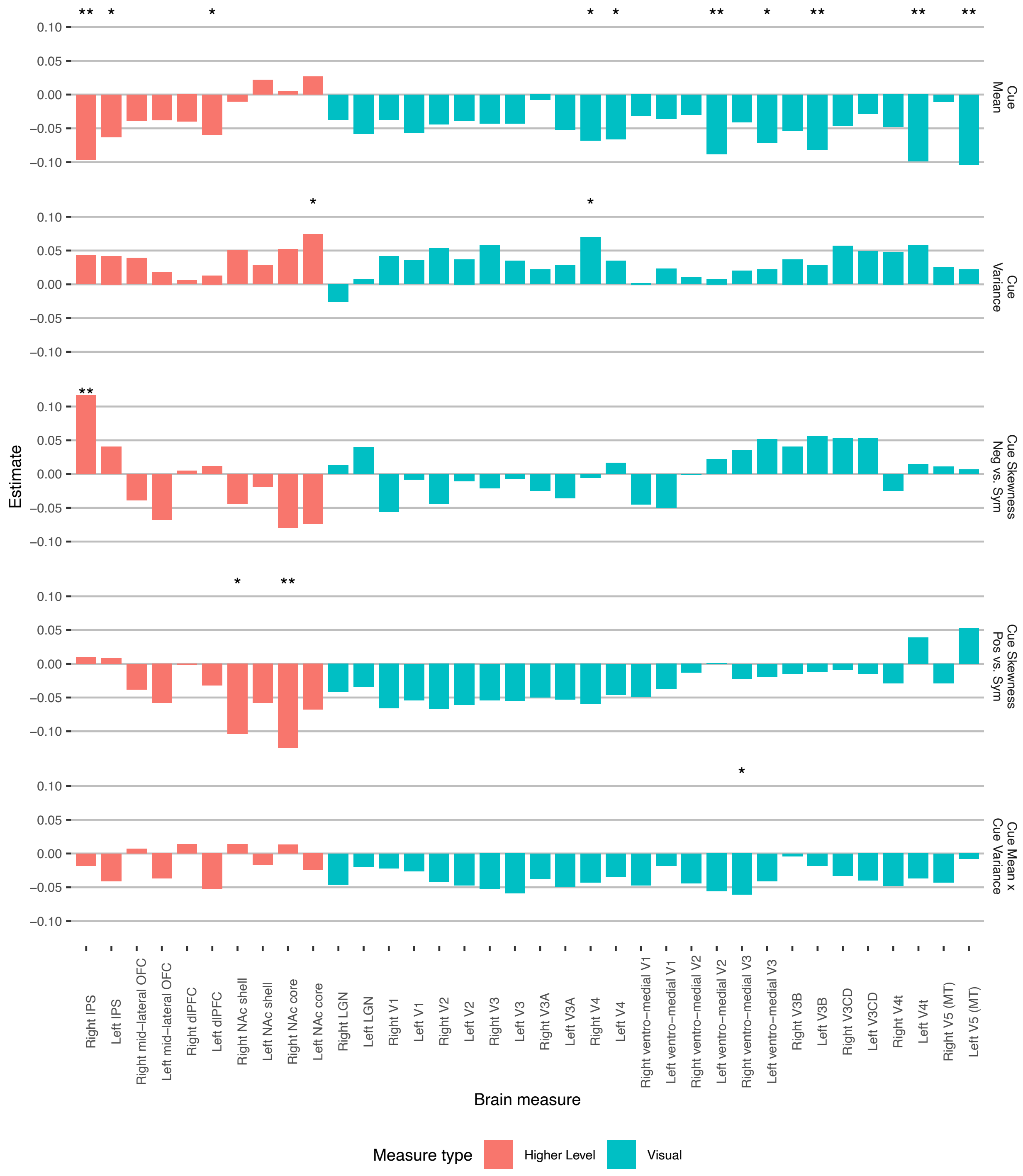

Supplementary Figure 4. Cue effects across all ROIs during anticipation for visual stimuli. Asterisks represent the level of significance (* $p < .05$, ** $p < .01$, *** $p < .001$). Abbreviations: Neg = negative; Pos = positive; Sym = symmetric; IPS = intraparietal sulcus; OFC = orbitofrontal cortex; dlPFC = dorsolateral prefrontal cortex; NAc = nucleus accumbens; LGN = lateral geniculate nucleus.

bioRxiv preprint doi: https://doi.org/10.1101/2024.10.10.617570; this version posted October 12, 2024. The copyright holder for this preprint (which was not certified by peer review) is the author/funder, who has granted bioRxiv a license to display the preprint in perpetuity. It is made available under aCC-BY 4.0 International license.

Supplementary Table 1. A priori regions of interest. Atlases are available via CANlab neuroimaging analysis tools (https://canlab.github.io/). The canlab2023 atlas is used with threshold = 0.2, and its regions included here are based on Glasser2016HCP atlas [1] for cortical regions, Tian2020 atlas [2] for the NAc, and Morel2010 atlas [3] for the thalamus.

Type	Region	Label
Early nociceptive pain processing (pain pathways atlas)	Dorsal posterior insula (dpIns)	dpIns_L, dpIns_R
	Ventral posterior thalamus	Thal_VPLM_L, Thal_VPLM_R
Nociceptive pain processing (pain pathways atlas)	Periaqueductal gray (PAG)	Bstem_PAG
	Anterior midcingulate cortex (aMCC)	aMCC_MPFC
	Medial thalamus	Thal_MD
Higher-level pain processing (canlab2023 atlas based on Glasser2016HCP atlas [1] for cortical regions and	Nucleus accumbens (NAc) - shell	NAc_shell_like_L, NAc_shell_like_R
	Nucleus accumbens (NAc) - core	NAc_core_like_L, NAc_core_like_R
	Mid lateral orbitofrontal cortex (OFC)	Ctx_111_L, Ctx_111_R
	dlPFC	Left: Ctx_8C_L and Ctx_46_L; Right: Ctx_p9_46v_R
Other pain processing (pain pathways atlas)	Primary somatosensory (S1), mostly hand area	s1_handplus_L, s1_handplus_R
	Anterior insula (aIns)	aIns_L, aIns_R
Early visual processing (canlab2023_coarse_2mm atlas)	Lateral geniculate nucleus (LGN)	Thal_Lateral_Geniculate_Nucleus_L, Thal_Lateral_Geniculate_Nucleus_R
	Primary visual cortex (V1)	Ctx_V1_L,Ctx_V1_R
	Secondary visual cortex (V2)	Ctx_V2_L,Ctx_V2_R
Visual processing (canlab2023_coarse_2mm atlas)	V3	Ctx_V3_L, Ctx_V3_R
	V4	Ctx_V4_L, Ctx_V4_R
	Middle temporal visual area V5 (MT)	Ctx_MT_L, Ctx_MT_R
Higher-level visual processing (canlab2023_coarse_2mm atlas)	Intraparietal sulcus (IPS)	Ctx_IPS1_L, Ctx_IPS1_R
Other visual processing (canlab2023_coarse_2mm atlas)	VMV1	Ctx_VMV1_L, Ctx_VMV1_R
	VMV2	Ctx_VMV2_L, Ctx_VMV2_R
	V3A	Ctx_V3A_L, Ctx_V3A_R
	V3B	Ctx_V3B_L, Ctx_V3B_R
	V3CD	Ctx_V3CD_L, Ctx_V3CD_R
	VMV3	Ctx_VMV3_L, Ctx_VMV3_R
	V4t	Ctx_V4t_L, Ctx_V4t_R

bioRxiv preprint doi: https://doi.org/10.1101/2024.10.10.617570; this version posted October 12, 2024. The copyright holder for this preprint (which was not certified by peer review) is the author/funder, who has granted bioRxiv a license to display the preprint in perpetuity. It is made available under aCC-BY 4.0 International license.

Supplementary Table 2. Statistics for the effects on NPS and SIIPS scores during visual perception.

Neuromarker	Effect	Estimate	SE	DF	t	p
NPS	Stimulus Intensity	-0.015	0.030	2947.1	-0.50	0.617
	Cue mean	-0.013	0.030	2946.4	-0.45	0.652
	Cue var	0.022	0.030	2947.9	0.75	0.456
	Cue SK (neg)	-0.055	0.042	2947.4	-1.29	0.196
	Cue SK (pos)	0.038	0.042	2947.3	0.90	0.368
	Cue mean x cue var	0.000	0.030	2946.4	0.02	0.988
SIIPS	Stimulus Intensity	-0.027	0.030	2935.7	-0.88	0.377
	Cue mean	-0.005	0.030	2935.1	-0.17	0.864
	Cue var	0.051	0.030	2936.7	1.68	0.094
	Cue SK (neg)	0.008	0.043	2936.1	0.20	0.844
	Cue SK (pos)	0.035	0.043	2935.9	0.81	0.417
	Cue mean x cue var	-0.011	0.030	2934.9	-0.38	0.704

bioRxiv preprint doi: https://doi.org/10.1101/2024.10.10.617570; this version posted October 12, 2024. The copyright holder for this preprint (which was not certified by peer review) is the author/funder, who has granted bioRxiv a license to display the preprint in perpetuity. It is made available under aCC-BY 4.0 International license.

Supplementary Table 3. Statistics for the effects on ROIs related to early pain perceptual processing. Abbreviations: var = variance; SK = skewness; neg = negative (vs. symmetric); pos = positive (vs. symmetric); stim int = stimulus intensity; VPL/VPM Thal = ventral posterior thalamus (medial and lateral); dpIns = dorsal posterior insula. Significant effects (uncorrected $p < .05$) are marked in bold.

Region	Effect	Estimate	SE	DF	t	p
Left dpIns	**Stimulus intensity**	**0.145**	**0.028**	**2906.0**	**5.17**	**< .001**
	Cue mean	-0.016	0.029	302.1	-0.55	0.585
	Cue var	0.002	0.028	2905.7	0.06	0.956
	Cue SK (neg)	0.030	0.040	2905.3	0.76	0.444
	Cue SK (pos)	0.005	0.040	2906.3	0.14	0.892
	Cue mean x cue var	-0.005	0.028	2913.9	-0.18	0.858
	Stim int x cue mean	-0.001	0.028	2904.7	-0.05	0.963
Left VPL/VPM Thal	**Stimulus intensity**	**0.084**	**0.029**	**2906.8**	**2.93**	**0.003**
	Cue mean	-0.043	0.029	314.4	-1.47	0.141
	Cue var	0.001	0.029	2906.6	0.05	0.962
	Cue SK (neg)	0.013	0.040	2906.1	0.31	0.757
	Cue SK (pos)	-0.005	0.040	2907.2	-0.13	0.898
	Cue mean x cue var	0.006	0.029	2914.8	0.22	0.830
	Stim int x cue mean	0.005	0.029	2905.6	0.17	0.862
Right dpIns	**Stimulus intensity**	**0.155**	**0.027**	**2948.3**	**5.67**	**< .001**
	Cue mean	-0.027	0.027	2948.6	-1.00	0.316
	Cue var	-0.019	0.027	2949.1	-0.68	0.499
	Cue SK (neg)	0.012	0.039	2948.6	0.31	0.754
	Cue SK (pos)	0.030	0.039	2948.8	0.78	0.435
	Cue mean x cue var	-0.003	0.027	2948.5	-0.10	0.916
	Stim int x cue mean	-0.007	0.027	2948.7	-0.24	0.808
Right VPL/VPM Thal	**Stimulus intensity**	**0.112**	**0.029**	**2946.5**	**3.88**	**< .001**
	Cue mean	-0.025	0.029	2946.9	-0.87	0.385
	Cue var	0.008	0.029	2947.7	0.28	0.783
	Cue SK (neg)	-0.031	0.041	2946.9	-0.75	0.456
	Cue SK (pos)	-0.045	0.041	2947.3	-1.10	0.273
	Cue mean x cue var	-0.006	0.029	2946.8	-0.21	0.836
	Stim int x cue mean	-0.027	0.029	2947.0	-0.94	0.346

bioRxiv preprint doi: https://doi.org/10.1101/2024.10.10.617570; this version posted October 12, 2024. The copyright holder for this preprint (which was not certified by peer review) is the author/funder, who has granted bioRxiv a license to display the preprint in perpetuity. It is made available under aCC-BY 4.0 International license.

Supplementary Table 4. Statistics for the effects on ROIs related to early visual perceptual processing. Abbreviations: var = variance; SK = skewness; neg = negative (vs. symmetric); pos = positive (vs. symmetric); stim int = stimulus intensity; LGN = lateral geniculate nucleus. Significant effects (uncorrected $p < .05$) are marked in bold.

Region	Effect	Estimate	SE	DF	t	p
Right LGN	Stimulus Intensity	0.024	0.031	2926.4	0.78	0.438
	Cue mean	0.013	0.031	308.5	0.41	0.683
	Cue var	0.012	0.031	2926.1	0.40	0.687
	Cue SK (neg)	0.009	0.043	2926.0	0.21	0.837
	Cue SK (pos)	0.010	0.043	2925.8	0.23	0.819
	Cue mean x var	0.012	0.031	2927.4	0.38	0.702
	Stim int x cue mean	-0.014	0.031	2927.1	-0.46	0.646
Left LGN	Stimulus Intensity	-0.030	0.031	2963.7	-0.97	0.332
	Cue mean	0.010	0.031	2962.8	0.34	0.736
	Cue var	0.011	0.031	2963.8	0.37	0.712
	Cue SK (neg)	0.027	0.044	2963.3	0.63	0.531
	Cue SK (pos)	-0.005	0.043	2963.7	-0.12	0.902
	Cue mean x var	0.025	0.031	2962.7	0.81	0.418
	Stim int x cue mean	-0.023	0.031	2962.8	-0.75	0.456
Right V1	**Stimulus Intensity**	**0.059**	**0.029**	**2947.0**	**2.04**	**0.041**
	Cue mean	-0.017	0.029	2946.4	-0.58	0.563
	Cue var	0.022	0.029	2947.0	0.78	0.438
	Cue SK (neg)	-0.054	0.041	2946.8	-1.32	0.187
	Cue SK (pos)	-0.009	0.041	2946.6	-0.23	0.821
	Cue mean x var	0.022	0.029	2946.3	0.75	0.456
	Stim int x cue mean	-0.032	0.029	2946.4	-1.11	0.269
Left V1	Stimulus Intensity	0.053	0.029	2951.0	1.83	0.067
	Cue mean	-0.037	0.029	2950.4	-1.27	0.204
	Cue var	-0.011	0.029	2951.0	-0.39	0.696
	Cue SK (neg)	-0.059	0.041	2950.8	-1.43	0.153
	Cue SK (pos)	-0.008	0.041	2950.6	-0.21	0.838
	Cue mean x var	0.027	0.029	2950.4	0.94	0.348
	Stim int x cue mean	-0.031	0.029	2950.4	-1.06	0.289
Right V2	Stimulus Intensity	0.054	0.028	2956.6	1.89	0.058
	Cue mean	-0.023	0.028	2956.1	-0.80	0.423
	Cue var	0.013	0.028	2956.5	0.46	0.646
	Cue SK (neg)	-0.072	0.040	2956.4	-1.80	0.072
	Cue SK (pos)	-0.034	0.040	2956.3	-0.84	0.401
	Cue mean x var	0.015	0.028	2956.0	0.51	0.608
	Stim int x cue mean	-0.021	0.028	2956.1	-0.74	0.461
Left V2	**Stimulus Intensity**	**0.064**	**0.029**	**2953.0**	**2.20**	**0.028**
	Cue mean	-0.032	0.029	2952.4	-1.09	0.275
	Cue var	0.003	0.029	2953.0	0.11	0.916
	Cue SK (neg)	-0.079	0.041	2952.8	-1.92	0.056
	Cue SK (pos)	-0.044	0.041	2952.6	-1.08	0.282
	Cue mean x var	0.025	0.029	2952.4	0.85	0.395
	Stim int x cue mean	-0.021	0.029	2952.4	-0.74	0.460

bioRxiv preprint doi: https://doi.org/10.1101/2024.10.10.617570; this version posted October 12, 2024. The copyright holder for this preprint (which was not certified by peer review) is the author/funder, who has granted bioRxiv a license to display the preprint in perpetuity. It is made available under aCC-BY 4.0 International license.

Supplementary Table 5. Statistics for the effects on ROIs related to pain perceptual processing. Abbreviations: var = variance; SK = skewness; neg = negative (vs. symmetric); pos = positive (vs. symmetric); stim int = stimulus intensity; aMCC = anterior midcingulate cortex; Med Thal = medial thalamus; PAG = periaqueductal gray. Significant effects (uncorrected $p < .05$) are marked in bold.

Region	Effect	Estimate	SE	DF	t	p
aMCC	**Stimulus Intensity**	**0.109**	**0.028**	**2905.4**	**3.92**	**< .001**
	Cue mean	-0.010	0.029	243.7	-0.35	0.728
	Cue var	0.001	0.028	2905.0	0.03	0.973
	Cue SK (neg)	0.031	0.039	2904.8	0.79	0.432
	Cue SK (pos)	0.040	0.039	2905.5	1.03	0.303
	Cue mean x var	-0.021	0.028	2912.4	-0.75	0.453
	Stim int x cue mean	-0.029	0.028	2904.1	-1.06	0.291
Med Thal	**Stimulus Intensity**	**0.097**	**0.029**	**2948.5**	**3.34**	**0.001**
	Cue mean	-0.005	0.029	2948.9	-0.16	0.872
	Cue var	-0.027	0.029	2949.8	-0.94	0.347
	Cue SK (neg)	0.017	0.041	2949.0	0.42	0.674
	Cue SK (pos)	-0.017	0.041	2949.4	-0.41	0.680
	Cue mean x var	-0.010	0.029	2948.8	-0.35	0.728
	Stim int x cue mean	-0.010	0.029	2949.1	-0.35	0.726
PAG	**Stimulus Intensity**	**0.141**	**0.030**	**2907.3**	**4.71**	**< .001**
	**Cue mean**	**-0.066**	**0.030**	**341.9**	**-2.17**	**0.031**
	Cue var	0.054	0.030	2907.8	1.79	0.073
	Cue SK (neg)	0.014	0.042	2906.8	0.33	0.739
	Cue SK (pos)	-0.001	0.042	2907.9	-0.04	0.972
	Cue mean x var	0.025	0.030	2915.3	0.83	0.406
	Stim int x cue mean	-0.028	0.030	2906.4	-0.92	0.357

bioRxiv preprint doi: https://doi.org/10.1101/2024.10.10.617570; this version posted October 12, 2024. The copyright holder for this preprint (which was not certified by peer review) is the author/funder, who has granted bioRxiv a license to display the preprint in perpetuity. It is made available under aCC-BY 4.0 International license.

Supplementary Table 6. Statistics for the effects on ROIs related to visual perceptual processing. Abbreviations: var = variance; SK = skewness; neg = negative (vs. symmetric); pos = positive (vs. symmetric); stim int = stimulus intensity; MT = middle temporal. Significant effects (uncorrected $p < .05$) are marked in bold.

Region	Effect	Estimate	SE	DF	t	p
Right V3	**Stimulus Intensity**	**0.055**	**0.027**	**2961.3**	**2.00**	**0.046**
	Cue mean	-0.021	0.027	2960.9	-0.77	0.439
	Cue var	0.024	0.027	2961.3	0.87	0.386
	**Cue SK (neg)**	**-0.102**	**0.039**	**2961.2**	**-2.63**	**0.009**
	Cue SK (pos)	-0.049	0.039	2961.1	-1.26	0.207
	Cue mean x var	-0.004	0.027	2960.9	-0.13	0.897
	Stim int x cue mean	-0.018	0.027	2960.9	-0.67	0.505
Left V3	**Stimulus Intensity**	**0.067**	**0.028**	**2955.9**	**2.39**	**0.017**
	Cue mean	-0.046	0.028	2955.5	-1.62	0.106
	Cue var	0.012	0.028	2955.9	0.42	0.672
	**Cue SK (neg)**	**-0.086**	**0.040**	**2955.7**	**-2.15**	**0.032**
	Cue SK (pos)	-0.062	0.040	2955.6	-1.54	0.123
	Cue mean x var	0.000	0.028	2955.4	-0.01	0.993
	Stim int x cue mean	-0.014	0.028	2955.4	-0.48	0.631
Right V4	Stimulus Intensity	0.035	0.027	2953.5	1.30	0.193
	Cue mean	-0.023	0.027	2953.2	-0.84	0.402
	Cue var	-0.009	0.027	2953.6	-0.32	0.747
	**Cue SK (neg)**	**-0.099**	**0.038**	**2953.5**	**-2.58**	**0.010**
	Cue SK (pos)	-0.061	0.038	2953.4	-1.59	0.111
	Cue mean x var	0.004	0.027	2953.2	0.13	0.894
	Stim int x cue mean	-0.005	0.027	2953.2	-0.18	0.857
Left V4	Stimulus Intensity	0.020	0.025	2959.5	0.81	0.421
	Cue mean	-0.032	0.025	2959.2	-1.28	0.201
	Cue var	-0.012	0.025	2959.5	-0.49	0.624
	**Cue SK (neg)**	**-0.072**	**0.036**	**2959.4**	**-2.01**	**0.045**
	Cue SK (pos)	-0.054	0.036	2959.3	-1.52	0.130
	Cue mean x var	0.006	0.025	2959.2	0.25	0.805
	Stim int x cue mean	0.008	0.025	2959.2	0.33	0.740
Right V5 (MT)	Stimulus Intensity	-0.003	0.029	2957.0	-0.10	0.922
	Cue mean	0.002	0.029	2956.5	0.06	0.954
	Cue var	-0.028	0.029	2957.0	-0.99	0.321
	Cue SK (neg)	-0.048	0.041	2956.9	-1.17	0.242
	Cue SK (pos)	-0.021	0.041	2956.8	-0.51	0.608
	Cue mean x var	0.027	0.029	2956.5	0.94	0.349
	Stim int x cue mean	-0.008	0.029	2956.5	-0.27	0.784
Left V5 (MT)	Stimulus Intensity	-0.018	0.029	2961.1	-0.63	0.531
	Cue mean	-0.015	0.029	2960.7	-0.51	0.607
	Cue var	-0.036	0.029	2961.2	-1.24	0.215
	**Cue SK (neg)**	**-0.091**	**0.041**	**2961.0**	**-2.22**	**0.027**
	Cue SK (pos)	-0.039	0.041	2960.9	-0.95	0.344
	Cue mean x var	0.012	0.029	2960.6	0.43	0.666
	Stim int x cue mean	0.044	0.029	2960.7	1.53	0.126

bioRxiv preprint doi: https://doi.org/10.1101/2024.10.10.617570; this version posted October 12, 2024. The copyright holder for this preprint (which was not certified by peer review) is the author/funder, who has granted bioRxiv a license to display the preprint in perpetuity. It is made available under aCC-BY 4.0 International license.

Supplementary Table 7. Statistics for the effects on ROIs related to higher level pain processing. Abbreviations: var = variance; SK = skewness; neg = negative (vs. symmetric); pos = positive (vs. symmetric); stim int = stimulus intensity; dlPFC = dorsolateral prefrontal cortex; OFC = orbitofrontal cortex; NAc = nucleus accumbens. Significant effects (uncorrected $p < .05$) are marked in bold.

Region	Effect	Estimate	SE	DF	t	p
Right dlPFC	Stimulus Intensity	0.021	0.03	2948.4	0.72	0.473
	Cue mean	-0.026	0.03	2948.8	-0.87	0.386
	Cue var	-0.04	0.03	2949.9	-1.34	0.181
	Cue SK (neg)	0.014	0.042	2949	0.34	0.737
	Cue SK (pos)	-0.007	0.042	2949.4	-0.17	0.868
	Cue mean x var	-0.005	0.03	2948.7	-0.18	0.854
	Stim int x cue mean	0.024	0.03	2949	0.81	0.417
Left dlPFC	**Stimulus Intensity**	**0.098**	**0.03**	**2948.5**	**3.31**	**0.001**
	Cue mean	-0.022	0.03	2948.9	-0.76	0.447
	Cue var	-0.03	0.03	2950	-1.02	0.307
	Cue SK (neg)	0.011	0.042	2949	0.26	0.798
	Cue SK (pos)	0.01	0.042	2949.5	0.25	0.801
	Cue mean x var	0.001	0.03	2948.8	0.05	0.961
	Stim int x cue mean	0.014	0.03	2949.1	0.48	0.631
Right mid-lateral OFC	Stimulus Intensity	-0.009	0.03	2948.5	-0.31	0.757
	Cue mean	0.017	0.03	2948.9	0.58	0.565
	Cue var	-0.049	0.03	2950	-1.64	0.102
	Cue SK (neg)	0.03	0.042	2949	0.72	0.469
	Cue SK (pos)	-0.011	0.042	2949.5	-0.26	0.793
	Cue mean x var	0.003	0.03	2948.8	0.11	0.916
	Stim int x cue mean	0.01	0.03	2949.1	0.32	0.748
Left mid-lateral OFC	Stimulus Intensity	-0.025	0.03	2904.5	-0.85	0.394
	Cue mean	-0.031	0.031	292.8	-1.02	0.308
	Cue var	-0.038	0.03	2904.6	-1.29	0.198
	Cue SK (neg)	0.019	0.042	2903.9	0.46	0.647
	Cue SK (pos)	0.002	0.042	2904.9	0.04	0.97
	Cue mean x var	0.023	0.03	2911.6	0.79	0.432
	Stim int x cue mean	0.028	0.03	2903.4	0.95	0.342
Right NAc core	**Stimulus Intensity**	**0.071**	**0.029**	**2904.9**	**2.39**	**0.017**
	**Cue mean**	**0.072**	**0.03**	**315.4**	**2.39**	**0.018**
	**Cue var**	**-0.075**	**0.029**	**2905**	**-2.53**	**0.011**
	Cue SK (neg)	-0.004	0.042	2904.3	-0.11	0.915
	Cue SK (pos)	-0.046	0.042	2905.3	-1.11	0.269
	Cue mean x var	0.047	0.029	2912.8	1.58	0.114
	Stim int x cue mean	-0.02	0.029	2903.8	-0.69	0.49
Right NAc shell	Stimulus Intensity	0.026	0.03	2907.9	0.87	0.384
	Cue mean	0.043	0.031	304.7	1.39	0.166
	Cue var	-0.052	0.03	2908.8	-1.73	0.084
	Cue SK (neg)	0.038	0.043	2907.7	0.88	0.378
	Cue SK (pos)	-0.005	0.043	2908.9	-0.11	0.909
	Cue mean x var	0.039	0.03	2915.3	1.31	0.192
	Stim int x cue mean	0.029	0.03	2907.1	0.95	0.344
Left NAc core	Stimulus Intensity	0.033	0.03	2947.9	1.1	0.273
	Cue mean	0.054	0.03	2948.4	1.81	0.07
	**Cue var**	**-0.104**	**0.03**	**2949.7**	**-3.47**	**0.001**
	Cue SK (neg)	0.019	0.042	2948.6	0.44	0.66
	Cue SK (pos)	-0.031	0.042	2949.1	-0.73	0.465
	**Cue mean x var**	**0.073**	**0.03**	**2948.3**	**2.43**	**0.015**
	Stim int x cue mean	-0.006	0.03	2948.6	-0.2	0.841
Right NAc core	Stimulus Intensity	0.045	0.03	2948.3	1.51	0.131
	Cue mean	0.021	0.03	2948.8	0.69	0.49
	Cue var	-0.02	0.03	2950.1	-0.67	0.5
	Cue SK (neg)	0.032	0.042	2948.9	0.76	0.445
	Cue SK (pos)	0.004	0.042	2949.5	0.09	0.929
	Cue mean x var	0.045	0.03	2948.6	1.5	0.133
	Stim int x cue mean	-0.002	0.03	2949	-0.06	0.956

bioRxiv preprint doi: https://doi.org/10.1101/2024.10.10.617570; this version posted October 12, 2024. The copyright holder for this preprint (which was not certified by peer review) is the author/funder, who has granted bioRxiv a license to display the preprint in perpetuity. It is made available under aCC-BY 4.0 International license.

Supplementary Table 8. Statistics for the effects on ROIs related to higher level visual processing. Abbreviations: var = variance; SK = skewness; neg = negative (vs. symmetric); pos = positive (vs. symmetric); stim int = stimulus intensity; IPS = intraparietal sulcus.

Region	Effect	Estimate	SE	DF	t	p
Left IPS	Stimulus Intensity	0.012	0.029	2958.7	0.40	0.691
	Cue mean	-0.054	0.029	2958.2	-1.84	0.066
	Cue var	-0.021	0.029	2958.8	-0.71	0.479
	Cue SK (neg)	-0.030	0.041	2958.6	-0.73	0.468
	Cue SK (pos)	-0.004	0.041	2958.5	-0.11	0.915
	Cue mean x var	0.029	0.029	2958.1	0.99	0.325
	Stim int x cue mean	-0.013	0.029	2958.1	-0.43	0.664
Right IPS	Stimulus Intensity	0.004	0.029	2956.7	0.14	0.891
	Cue mean	-0.052	0.029	2956.2	-1.79	0.074
	Cue var	-0.036	0.029	2956.8	-1.24	0.216
	Cue SK (neg)	-0.013	0.042	2956.6	-0.31	0.753
	Cue SK (pos)	0.000	0.041	2956.5	-0.01	0.991
	Cue mean x var	0.054	0.029	2956.2	1.86	0.064
	Stim int x cue mean	-0.015	0.029	2956.3	-0.52	0.605

bioRxiv preprint doi: https://doi.org/10.1101/2024.10.10.617570; this version posted October 12, 2024. The copyright holder for this preprint (which was not certified by peer review) is the author/funder, who has granted bioRxiv a license to display the preprint in perpetuity. It is made available under aCC-BY 4.0 International license.

Supplementary Table 9. Statistics for the effects on other pain processing ROIs. Abbreviations: var = variance; SK = skewness; neg = negative (vs. symmetric); pos = positive (vs. symmetric); stim int = stimulus intensity; aIns = anterior insula. Significant effects (uncorrected $p < .05$) are marked in bold.

Region	Effect	Estimate	SE	DF	t	p
Right aIns	**Stimulus Intensity**	**0.076**	**0.028**	**2904.4**	**2.68**	**0.007**
	Cue mean	0.018	0.029	295.0	0.61	0.540
	Cue var	0.008	0.028	2904.1	0.29	0.773
	Cue SK (neg)	0.007	0.040	2903.7	0.18	0.858
	Cue SK (pos)	0.071	0.040	2904.8	1.78	0.075
	Cue mean x var	0.002	0.028	2912.1	0.07	0.942
	Stim int x cue mean	-0.040	0.028	2903.2	-1.41	0.158
Left aIns	**Stimulus Intensity**	**0.093**	**0.029**	**2948.3**	**3.21**	**0.001**
	Cue mean	-0.016	0.029	2948.7	-0.54	0.588
	Cue var	0.002	0.029	2949.6	0.06	0.951
	Cue SK (neg)	0.024	0.041	2948.8	0.57	0.567
	Cue SK (pos)	0.045	0.041	2949.1	1.08	0.278
	Cue mean x var	-0.007	0.029	2948.6	-0.23	0.820
	Stim int x cue mean	-0.010	0.029	2948.9	-0.33	0.739
Right S1 hand	**Stimulus Intensity**	**0.117**	**0.026**	**2948.2**	**4.49**	**0.000**
	Cue mean	-0.007	0.026	2948.4	-0.27	0.788
	Cue var	-0.009	0.026	2948.8	-0.33	0.738
	Cue SK (neg)	0.015	0.037	2948.4	0.40	0.689
	Cue SK (pos)	0.036	0.037	2948.6	0.99	0.324
	Cue mean x var	0.018	0.026	2948.4	0.67	0.500
	Stim int x cue mean	-0.010	0.026	2948.5	-0.37	0.709
Left S1 hand	**Stimulus Intensity**	**0.133**	**0.027**	**2908.5**	**5.00**	**0.000**
	Cue mean	-0.033	0.027	357.6	-1.21	0.225
	Cue var	-0.012	0.027	2907.8	-0.46	0.645
	Cue SK (neg)	0.034	0.038	2907.6	0.90	0.370
	Cue SK (pos)	0.024	0.038	2908.5	0.63	0.527
	Cue mean x var	-0.020	0.027	2916.3	-0.76	0.445
	Stim int x cue mean	-0.009	0.027	2907.2	-0.34	0.736

bioRxiv preprint doi: https://doi.org/10.1101/2024.10.10.617570; this version posted October 12, 2024. The copyright holder for this preprint (which was not certified by peer review) is the author/funder, who has granted bioRxiv a license to display the preprint in perpetuity. It is made available under aCC-BY 4.0 International license.

Supplementary Table 10. Statistics for the effects on other visual processing ROIs. Abbreviations: var = variance; SK = skewness; neg = negative (vs. symmetric); pos = positive (vs. symmetric); stim int = stimulus intensity. Significant effects (uncorrected *p* < .05) are marked in bold.

Region	Effect	Estimate	SE	DF	t	p
Right V3A	Stimulus Intensity	0.041	0.027	2958	1.54	0.124
	Cue mean	-0.014	0.027	2957.7	-0.54	0.590
	Cue var	0.014	0.027	2958	0.54	0.590
	**Cue SK (neg)**	**-0.087**	**0.038**	**2957.9**	**-2.30**	**0.021**
	Cue SK (pos)	-0.040	0.038	2957.9	-1.05	0.292
	Cue mean x var	0.000	0.027	2957.7	0.02	0.987
	Stim int x cue mean	-0.003	0.027	2957.7	-0.11	0.911
Left V3A	Stimulus Intensity	0.043	0.028	2955.5	1.57	0.115
	Cue mean	-0.012	0.028	2955.2	-0.45	0.653
	Cue var	-0.003	0.028	2955.5	-0.10	0.920
	**Cue SK (neg)**	**-0.101**	**0.039**	**2955.4**	**-2.57**	**0.010**
	Cue SK (pos)	-0.052	0.039	2955.3	-1.33	0.184
	Cue mean x var	-0.022	0.028	2955.1	-0.82	0.415
	Stim int x cue mean	-0.004	0.028	2955.2	-0.13	0.898
Right V3B	Stimulus Intensity	0.033	0.029	2961.9	1.13	0.260
	Cue mean	-0.046	0.029	2961.4	-1.58	0.114
	Cue var	-0.013	0.029	2962	-0.44	0.657
	Cue SK (neg)	-0.046	0.041	2961.8	-1.13	0.260
	Cue SK (pos)	-0.029	0.041	2961.7	-0.71	0.476
	Cue mean x var	0.040	0.029	2961.4	1.39	0.164
	Stim int x cue mean	0.012	0.029	2961.4	0.42	0.672
Left V3B	Stimulus Intensity	0.007	0.028	2960.9	0.24	0.811
	Cue mean	-0.019	0.028	2960.5	-0.68	0.494
	Cue var	-0.024	0.028	2960.8	-0.88	0.379
	Cue SK (neg)	-0.055	0.039	2960.7	-1.40	0.163
	Cue SK (pos)	-0.023	0.039	2960.6	-0.58	0.562
	Cue mean x var	-0.003	0.028	2960.4	-0.11	0.911
	Stim int x cue mean	0.018	0.028	2960.5	0.64	0.523
Right V3CD	Stimulus Intensity	0.032	0.026	2946.2	1.23	0.219
	Cue mean	-0.022	0.026	2946	-0.85	0.393
	Cue var	-0.046	0.026	2946.2	-1.76	0.079
	Cue SK (neg)	-0.064	0.037	2946.2	-1.74	0.083
	Cue SK (pos)	-0.052	0.037	2946.1	-1.41	0.160
	Cue mean x var	0.031	0.026	2946	1.19	0.234
	Stim int x cue mean	0.021	0.026	2946	0.81	0.416
Left V3CD	Stimulus Intensity	0.001	0.026	2960.6	0.06	0.955
	Cue mean	-0.015	0.026	2960.3	-0.56	0.575
	Cue var	-0.038	0.026	2960.6	-1.48	0.139
	Cue SK (neg)	-0.020	0.037	2960.5	-0.54	0.588
	Cue SK (pos)	-0.035	0.037	2960.4	-0.94	0.346
	Cue mean x var	0.013	0.026	2960.3	0.51	0.609
	Stim int x cue mean	-0.008	0.026	2960.3	-0.30	0.765
Right V4t	Stimulus Intensity	0.001	0.029	2952.9	0.04	0.968
	Cue mean	0.008	0.029	2952.4	0.26	0.792
	Cue var	-0.021	0.029	2952.9	-0.73	0.466
	Cue SK (neg)	-0.062	0.040	2952.7	-1.52	0.128
	Cue SK (pos)	-0.042	0.040	2952.6	-1.05	0.293
	Cue mean x var	0.048	0.029	2952.4	1.67	0.095
	Stim int x cue mean	0.002	0.029	2952.4	0.05	0.957
Left V4t	Stimulus Intensity	-0.002	0.027	2961.3	-0.07	0.945
	Cue mean	-0.022	0.027	2961.1	-0.84	0.398
	Cue var	-0.041	0.027	2961.4	-1.54	0.125
	Cue SK (neg)	-0.062	0.038	2961.3	-1.64	0.101
	Cue SK (pos)	-0.056	0.038	2961.2	-1.48	0.138
	Cue mean x var	0.020	0.027	2961.1	0.73	0.463
	Stim int x cue mean	0.026	0.027	2961.1	0.97	0.334

bioRxiv preprint doi: https://doi.org/10.1101/2024.10.10.617570; this version posted October 12, 2024. The copyright holder for this preprint (which was not certified by peer review) is the author/funder, who has granted bioRxiv a license to display the preprint in perpetuity. It is made available under aCC-BY 4.0 International license.

Right ventro-medial V1	Stimulus Intensity	0.023	0.030	2955.6	0.79	0.432
	Cue mean	0.020	0.030	2954.8	0.66	0.510
	Cue var	-0.013	0.030	2955.6	-0.43	0.668
	Cue SK (neg)	-0.035	0.042	2955.4	-0.82	0.413
	Cue SK (pos)	-0.012	0.042	2955.2	-0.28	0.780
	Cue mean x var	-0.018	0.030	2954.7	-0.61	0.542
	Stim int x cue mean	0.005	0.030	2954.9	0.18	0.856
Left ventro-medial V1	Stimulus Intensity	0.007	0.030	2915.6	0.25	0.806
	Cue mean	-0.017	0.031	330.5	-0.55	0.585
	Cue var	0.033	0.030	2915	1.09	0.275
	Cue SK (neg)	-0.026	0.043	2915.2	-0.62	0.537
	Cue SK (pos)	0.005	0.043	2914.6	0.12	0.903
	Cue mean x var	-0.017	0.030	2916.3	-0.56	0.577
	Stim int x cue mean	0.014	0.030	2917.3	0.48	0.632
Right ventro-medial V2	Stimulus Intensity	0.018	0.030	234.8	0.58	0.564
	Cue mean	-0.005	0.030	266.1	-0.18	0.857
	Cue var	0.013	0.029	2875.1	0.45	0.650
	Cue SK (neg)	-0.068	0.040	2874.7	-1.68	0.094
	Cue SK (pos)	-0.039	0.040	2880.5	-0.96	0.339
	Cue mean x var	-0.026	0.029	2875.7	-0.91	0.362
	Stim int x cue mean	-0.005	0.029	2877.1	-0.16	0.874
Left ventro-medial V2	Stimulus Intensity	-0.021	0.029	2959.2	-0.73	0.466
	Cue mean	-0.026	0.029	2958.7	-0.91	0.360
	Cue var	-0.020	0.029	2959.2	-0.70	0.486
	Cue SK (neg)	-0.006	0.041	2959	-0.16	0.876
	Cue SK (pos)	-0.024	0.041	2958.9	-0.58	0.564
	Cue mean x var	-0.011	0.029	2958.6	-0.38	0.702
	Stim int x cue mean	-0.008	0.029	2958.7	-0.26	0.793
Right ventro-medial V3	Stimulus Intensity	0.033	0.028	2911	1.16	0.247
	Cue mean	-0.014	0.029	319.8	-0.48	0.629
	Cue var	0.016	0.028	2910.1	0.55	0.582
	Cue SK (neg)	-0.078	0.040	2910.3	-1.93	0.053
	Cue SK (pos)	-0.046	0.040	2910.5	-1.15	0.249
	Cue mean x var	0.000	0.028	2912.5	0.02	0.987
	Stim int x cue mean	-0.014	0.028	2913	-0.49	0.624
Left ventro-medial V3	Stimulus Intensity	0.027	0.028	2962.7	0.98	0.326
	Cue mean	-0.027	0.028	2962.5	-0.97	0.330
	Cue var	-0.012	0.028	2962.8	-0.45	0.651
	Cue SK (neg)	-0.057	0.039	2962.8	-1.45	0.147
	Cue SK (pos)	-0.033	0.039	2962.6	-0.84	0.403
	Cue mean x var	0.006	0.028	2962.4	0.23	0.819
	Stim int x cue mean	-0.019	0.028	2962.5	-0.70	0.482

bioRxiv preprint doi: https://doi.org/10.1101/2024.10.10.617570; this version posted October 12, 2024. The copyright holder for this preprint (which was not certified by peer review) is the author/funder, who has granted bioRxiv a license to display the preprint in perpetuity. It is made available under aCC-BY 4.0 International license.

References (Supplementary Information)

1. Glasser, M. F. *et al.* A multi-modal parcellation of human cerebral cortex. *Nature* **536**, 171–178 (2016).
2. Tian, Y., Margulies, D. S., Breakspear, M. & Zalesky, A. Topographic organization of the human subcortex unveiled with functional connectivity gradients. *Nat. Neurosci.* **23**, 1421–1432 (2020).
3. Krauth, A. *et al.* A mean three-dimensional atlas of the human thalamus: generation from multiple histological data. *Neuroimage* **49**, 2053–2062 (2010).
